# Protocol and rationale for the first South Asian 5-year prospective longitudinal observational cohort study and biomarker evaluation investigating the clinical course and risk profile of IgA nephropathy: GRACE IgANI cohort

**DOI:** 10.12688/wellcomeopenres.14644.1

**Published:** 2018-07-26

**Authors:** Suceena Alexander, George T. John, Anila Korula, T. S. Vijayakumar, Vinoi George David, Anjali Mohapatra, Anna T. Valson, Shibu Jacob, Pradeep Mathew Koshy, Gautam Rajan, Elenjickal Elias John, Smita Mary Matthai, L. Jeyaseelan, Babu Ponnusamy, Terence Cook, Charles Pusey, Mohamed R. Daha, John Feehally, Jonathan Barratt, Santosh Varughese

**Affiliations:** 1 Department of Nephrology, Christian Medical College, Vellore, Tamil Nadu, 632004, India; 2 Department of Renal Medicine, Royal Brisbane and Women's Hospital, Queensland, 4029, Australia; 3 Department of General Pathology, Christian Medical College, Vellore, Tamil Nadu, 632004, India; 4 Central Electron Microscope Unit, Christian Medical College, Vellore, Tamil Nadu, 632004, India; 5 Department of Biostatistics, Christian Medical College, Vellore, Tamil Nadu, 632004, India; 6 Centre for Cellular and Molecular Platforms, Bengaluru, Karnataka, 560065, India; 7 Centre for Complement and Inflammation Research, Imperial College, London, UK; 8 Department of Medicine, Imperial College Healthcare NHS Trust, London, UK; 9 Rijksuniversiteit Groningen Faculteit Biologie, Groningen, Netherlands; 10 University of Leicester, College of Medicine Biological Sciences and Psychology, Leicester, UK

**Keywords:** Immunology, Nephrology, IgA nephropathy, Glomerulonephritis, Pathology, South-Asians, Indians, Epidemiology, Protocol

## Abstract

**Background:** IgA nephropathy (IgAN) is the most common primary glomerulonephritis and an important cause of end-stage kidney disease. Unlike the slowly progressive course seen among Caucasian and East Asian subjects (actuarial survival 80-85% over 10 years), in India about 30-40% of patients have nephrotic syndrome and renal dysfunction at presentation and a 10-year renal survival of 35%, as reported from a retrospective registry. These observations cannot be entirely attributed to a lack of uniform screening protocols or late referral and attest to the probability that IgAN may not be the same disease in different parts of the world.

**Methods:** We will prospectively recruit 200 patients with IgAN (the GRACE IgANI— **G**lomerular **R**esearch **A**nd **C**linical **E**xperiments- **I**g **A** **N**ephropathy in **I**ndians—cohort) and stratify them into low and high risk of progression based on published absolute renal risk scores. We will test the validity of this risk score in an unselected Indian IgAN population over a 5-year follow-up period. In parallel, we will undertake extensive exploratory serum, urine, renal and microbiome biomarker studies, firstly, to determine if the underlying pathogenic pathways are the same in Indian IgAN compared to those reported in Caucasian and East Asian IgAN. Secondly, we will systematically assess the value of measuring selected biomarkers and adding this data to traditional measures of risk in IgAN to predict kidney failure. We ultimately hope to generate a composite IgAN risk score specific for the Indian population.

**Ethics and data dissemination:** Approval was obtained from the Institutional Review Board (Silver, Research and Ethics Committee) of the Christian Medical College, Vellore, India (Ref. No. IRB Min. No. 8962 [Other] dated 23.07.2014 and IRB Min. No. 9481 [Other] dated 24.06.2015). It is anticipated that results of this study will be presented at national and international meetings, with reports being published from late 2018.

## Introduction

### Global burden of IgA Nephropathy (IgAN)

IgAN is a frequent cause of end-stage kidney disease (ESKD) in both White and Asian populations. By contrast, IgAN has rarely been detected in Black populations, either from the United States or Africa. The prognosis of patients with normal renal function and proteinuria <1 g per day is excellent, with one study reporting 98% renal survival over 15 years ^1^. By contrast, 15–25% of patients develop ESKD over 20 years if there is significant proteinuria (>1 g per day) and existing evidence of renal dysfunction ^2^.

### What is known in South Asia and India

Incidence of glomerular disease in the tropics is much greater than in temperate countries. Nephrotic syndrome is 60–100 times more common in India than in the UK and USA ^3^. Glomerular diseases are often a consequence of interaction between an environmental nephritogenic antigen and a genetically susceptible host. Hence, the high rate of antigenic exposure in the tropics could account for this high incidence of glomerulonephritis. There is no national glomerulonephritis registry in India. Most epidemiological studies are single- or multi-centre retrospective analyses of renal biopsy datasets.

The most common presentation of primary glomerulonephritis in various studies in India is nephrotic syndrome and renal impairment ( Table 1). Asymptomatic urinary abnormalities are reported far less frequently (1.7–9%) ^4, 5^ when compared to data from registries in other developed nations like Japan and Italy ^6, 7^. This is perhaps not surprising as there is no universal urine screening program in India, precluding detection of the disease at an early asymptomatic stage. IgAN is the most common primary glomerulonephritis in India, identified in 10–15% of all renal biopsies reported from our institution and elsewhere in India ^4, 5, 8, 9^. Retrospective studies from renal registries in India report that 30–40% of patients with IgAN have nephrotic syndrome and renal dysfunction at presentation ^4, 9– 11^. Indian patients present with IgAN approximately over a decade earlier than Caucasian and East Asian patients, whose average age at presentation is 35–40 years ^12– 14^. These observations cannot be entirely attributed to differences in access to primary care and country-specific screening programs for kidney disease, and attest to the likelihood that IgAN may not be the same disease in different parts of the world.

**Table 1.** Renal biopsy studies from India.

Studies	Renal biopsies, N	Period, years	IgAN, n (%)	Nephrotic syndrome, %	Nephritic syndrome (%)	Hypertension, %	Renal dysfunction, %
Bhuyan ^8^ (1992) (Delhi, North India)	1146		83 (7.2)	24	NA	39	34
Sehgal ^16^ (1995) (Chandigarh, North India)	106	-	11 (10.4)	11	NA	NA	NA
Muthukumar ^11^ (2002) (Chennai, South India)	NA*		98 (NA)	25.6	5.1	9.2	13.5
Narasimhan ^4, 17^ (2006) (CMC Vellore, South India)	5415	1986–2002	478 (8.6)	55	16	58	60
Vanikar ^18^ (2005) (Gujarat, Western India)	4132	1998–2004	120 (16.2)	NA	NA	NA	NA
Chandrika ^19^ (2007) (Kerala, South India)	1592	2 years	227 (14.3)	36.7	18.9	3.5	5.7
Das ^5^ (2011) (Hyderabad, Central India)	1849	1990–2008	81 (4.4)	44.4	21	65.4	39
Siddappa ^9^ (2013) (Mangalore, South India)	400	2007–2010	31 (7.8)	35.5	NA	45.2	38.7
Jeganathan ^20^ (2013) Mangalore, South India	75	2 years	12 (16)	0	83.3	16.7	0
Golay ^21^ (2013) Eastern India	666	2010–2012	54 (8.11)	6.09	9.23	-	10

NA, not applicable.

George *et al*. ^15^, at Christian Medical College, Vellore, India (CMC Vellore), reported IgAN in 9.6% of 649 adults with primary glomerulonephritis. Hypertension was detected in 51.6% of patients and renal failure in 32.3%. Follow up was possible in 61.3% for a mean period of 17.3 months and progression to ESKD was noted in 7.9% ^15^. Muthukumar *et al.* (Chennai, South India) showed in their cohort of 98 patients that five-year renal survival was 38.5% (CI 24.6%–52.3%) ^11^.

**Figure 1.**
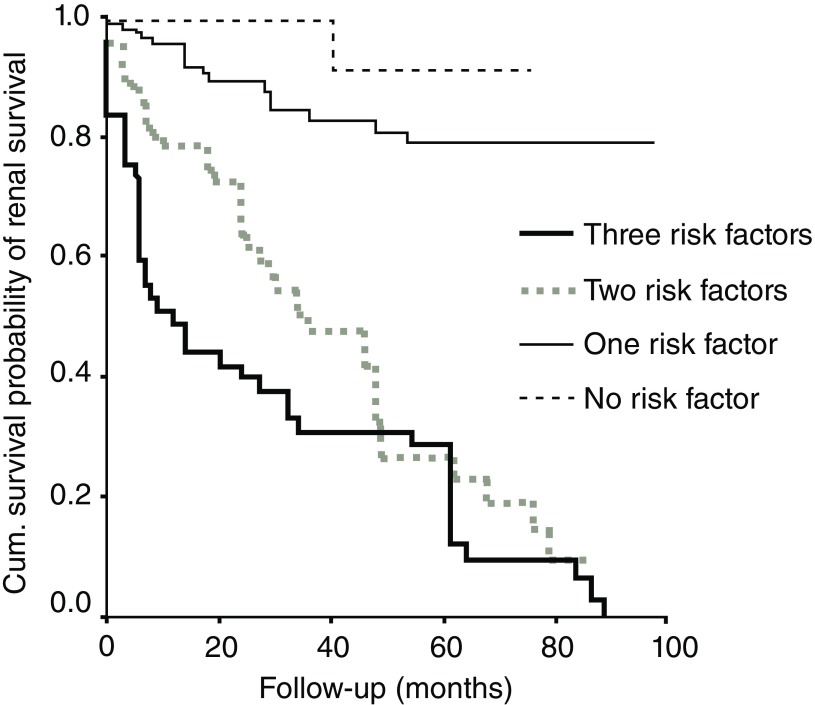
IgA nephropathy in India is associated with poorer renal survival. Risk factors: hypertension, proteinuria, interstitial fibrosis, sclerosed glomeruli. Figure has been reproduced with permission from Chacko *et al.* ^23^ under a CC-BY-NC-SA license.

Chacko *et al*. (CMC Vellore) reported that four factors were significantly associated with ESKD. These were hypertension, nephrotic range proteinuria, degree of sclerosed glomeruli, and interstitial fibrosis at presentation. The mean renal survival time of patients without any of these was 132 months, whereas those with one, two, or three risk factors were 105, 47, and 28 months, respectively ( Figure 1). A total of 46% of patients either progressed to ESKD or had permanent decline in renal function at last follow-up. Overall renal survival at 10 years was only 35% when compared with nearly 90% in similar studies from Singapore, Australia, and France. Also, patients diagnosed from the year 2000 onward had poorer outcomes, than those diagnosed before the year 2000 ^22, 23^.

### Traditional prognostic factors in IgAN

Persistent proteinuria, hypertension, and an increased serum creatinine level at presentation are the strongest clinical predictors of progression in IgAN ^1, 2, 24^. The Oxford histological classification of IgAN was developed by the International IgA Nephropathy Network in collaboration with the Renal Pathology Society in 2009 ^25, 26^. Retrospective analysis of renal biopsies from 265 patients identified four variables that, independent of one another and clinical features, were correlated with renal outcome: mesangial hypercellularity, **s**egmental sclerosis, **e**ndocapillary hypercellularity and **t**ubular atrophy/interstitial fibrosis (MEST) ^25, 26^. The prognostic power of the Oxford classification has subsequently been validated in a number of different IgAN cohorts from across the globe ^27– 29^. In 2017, the Oxford classification was amended to include a C (MEST-C) score to highlight the significance of crescents in IgAN ^30^.

A prediction score called the ‘absolute renal risk score’ (ARR), uses clinical measures and the Oxford classification to estimate the 5-year risk of developing ESKD in IgAN ^31^. This risk score was developed by Berthoux ^32^ and validated in Japan with a cohort of 702 IgAN patients ^31^, but has not been studied in an Indian population, who have a more rapid rate of renal decline.

### The pathogenesis of IgAN: A Caucasian perspective

Studies of IgAN have consistently identified changes in the *O*-glycans attached to the hinge region of the IgA1 immunoglobulin heavy chain ^33– 35^. These changes are present in both serum and mesangial IgA1 ^34^. This reduction in the number of *O*-linked galactose residues at the IgA1 hinge has been detected using specific lectin binding assays, fluorophore-assisted carbohydrate electrophoresis and mass spectrometry ^36^. These IgA1 molecules have been shown to activate human mesangial cells *in vitro* ^37^ resulting in the generation of IgG and IgA1 anti-hinge region autoantibodies and circulating immune complexes that leads to activation of the complement system through the lectin and alternative pathways ^38, 39^. Patients with IgAN have higher levels of poorly galactosylated IgA1 in their serum than healthy subjects ^40, 41^, and these levels appear to correlate with a higher likelihood of developing progressive renal failure ^42^. In a study of 97 patients with IgAN of varying severity, titres of IgG anti-hinge region autoantibodies correlated with both the ARR and the risk of ESKD or death ^43^. There is also an emerging role for circulating secretory IgA in IgAN, with recent studies reporting that secretory IgA can bind to and activate human mesangial cells more strongly than non-secretory IgA ^44, 45^. Recent data show that IgAN patients have altered fecal microbiota and volatile organic compounds (VOC) profiling, which in turn differed between progressors and non-progressors ^46^.

There is clear evidence of complement activation within the glomerulus in IgAN, with 60–70% of all IgAN biopsies demonstrating C3 deposition in the mesangium. There is evidence from both immunohistochemical and genetic studies for both activation of the alternative and lectin pathways in IgAN, but little evidence for classical pathway activation ^38, 39^. Roos *et al.* examined 60 renal biopsies of IgAN patients and found mannose binding lectin (MBL), a key component of the lectin pathway, co-deposition in 15 (25%) of cases. MBL deposition was associated with more severe renal disease ^47^.

In this study, we will assess the correlation between clinical and histological features, and measures of immunological activation in a cohort of Indian patients with IgAN. We will use our understanding of the pathogenesis of IgAN in Caucasians and East Asians to determine whether factors such as serum levels of secretory IgA, IgA immune complexes and IgG anti-hinge region autoantibodies can be used to risk stratify patients and monitor response to immunotherapy in an Indian population.

The Kidney Disease Improving Global Outcomes (KDIGO) guidelines on glomerulonephritis ^48^ do not recommend immunosuppression in most patients with IgAN. However, considering the severe presentation and progression of IgAN in India, the standard protocol for managing patients with IgAN at CMC Vellore is to treat with immunosuppression in those who have a urinary protein excretion >1 g/day with/without renal dysfunction along maximally tolerated inhibition of the renin-angiotensin system (RAS). We give oral prednisolone at 2 mg/kg alternate days (max. 120 mg) for 3 months and then taper and stop over the next 3 months. Mycophenolic acid is added as a second immunosuppressor at 25–30 mg/kg, with monitoring of area under the curve (target 30–50 mg.h/l), if they are persistently nephrotic with or without renal dysfunction and if the therapy is affordable to the patient. The treatment dilemma in IgAN has been highlighted recently ^49^ and we will carefully monitor the effects of immunosuppression in this cohort.

## Protocol

We hypothesise that the natural history and risk of renal progression in Indian patients with IgAN differs from that widely reported for Caucasian and East Asian patients. This may be due to differences in the underlying pathogenic pathways operating in Indian patients. A completed STROBE reporting checklist is available ( Supplementary File 1).

## Primary objective

- To measure exploratory biomarkers in an incident Indian population with IgAN and look for associations with clinical outcomes at 2 and 5 years of follow-up.

## Secondary objectives

- To measure exploratory biomarkers in an incident Indian population of IgAN and compare these findings with biomarker studies in Caucasian and East Asian IgAN populations to determine whether pathogenic pathways are similar across ethnicities.
- To determine the impact of immunosuppressive agents on the levels of selected biomarkers in an Indian IgAN population.
- To develop renal risk score for Indian patients with IgAN to help counsel patients at the time of diagnosis and direct health resources and immunosuppressive therapy to those patients at greatest risk of ESKD.

## Patient recruitment

The study will be conducted in the Department of Nephrology, CMC Vellore. CMC Vellore is the largest private not-for-profit tertiary referral hospital in South India and caters to patients mainly from South, North and North-East India. All patients being planned for a renal biopsy at CMC Vellore will be screened and all eligible patients will be approached to participate in the study. The inclusion and exclusion criteria are given in Table 2. These criteria apply to all IgAN and non-IgAN participants. All consenting patients will be recruited prior to the renal biopsy procedure. IgA nephropathy will be diagnosed by the presence of IgA-dominant or co-dominant immune deposits within glomeruli, as shown by immunofluorescent staining of the renal biopsy tissue ^25^. The non-IgAN kidney disease control arm will be patients with biopsy-proven primary glomerulonephritis other than IgAN. The healthy control group will be matched for age and gender to the IgAN cohort and will be voluntary kidney donors on work-up for kidney donation. Both arms will be matched in terms of sample size. The Caucasian and East Asian patient cohorts will be obtained from the University of Leicester, IgA nephropathy lab database, and the main collaborator for this work is J.B. Details concerning enrollment and ethical approval of this arm have been previously published ^50^. Some of the initial biomarkers we propose to test in sera are levels of galactose-deficient IgA1, immune complexes, antibody to galactose-deficient IgA1, secretory IgA, immune cell markers among other exploratory biomarkers to be determined during the study. The renal tissue will also be stained for components of the alternative and lectin pathways and for IgA1 components. The study started patient recruitment in March 2015 and ended at the last quarter of 2017. All patients are being followed up prospectively.

**Table 2.** Inclusion and exclusion criteria for screening patient eligibility prior to recruitment into the GRACE-IgANI cohort study.

Inclusion criteria	Exclusion criteria
Age ≥18 years	Secondary IgA nephropathy: e.g. due to lupus, liver cirrhosis, Henoch-Schonlein purpura.
Primary IgAN diagnosed by renal biopsy	Glomerular filtration rate as estimated by the CKD-EPI equation <10 ml/min/1.73 m ^2^.
Immunosuppression naive for three months prior to recruitment	Patients with systemic diseases that can affect the kidneys like diabetes, systemic lupus erythematosus, presence of HIV, HBsAg, HCV infections, malignancies etc.
Willing to come for follow-up visits	Patients with a history of psychological illness or condition which interferes with their ability to understand or comply with the requirements of the study.

IgAN, IgA nephropathy; HBsAg, hepatitis B surface antigen; HCV, hepatitis C virus.

## Outcome measures

### Primary outcome

The primary outcome is the evaluation of a range of aforementioned exploratory serum, urine and renal biomarkers and the investigation of their association to the rate of fall in estimated glomerular filtration rate (eGFR) at 2 and 5 years in a low- and high-risk incident Indian IgAN population as defined by ARR scores of <23 and of ≥23 points, respectively, at baseline and for the whole cohort.

### Secondary outcomes

1. Evaluation of a range of exploratory serum, urine and renal biomarkers, and to investigate their association to a composite end-point of at-least 50% decline in eGFR and ESKD (eGFR <15 ml/min/1.73 m ^2^), renal replacement therapy (RRT) or death at 2 and 5 years in a low- and high-risk incident Indian IgAN population as defined by an ARR score of <23 and of ≥23 points, respectively, at baseline and for the whole cohort.
2. To determine the validity of the ARR to predict either the rate of fall in eGFR or a composite end-point of at least 50% decline in eGFR and ESKD (eGFR <15 ml/min/1.73 m ^2^), RRT or death at 2 and 5 years in an unselected incident Indian population with IgAN.
3. To compare exploratory biomarkers in an incident Indian population of IgAN and compare these findings with biomarker studies in Caucasian and East Asian IgAN populations to determine whether pathogenic pathways are similar across ethnicities.
4. To determine whether the addition of novel biomarker measurements can improve the predictive performance of an RRS in an Indian IgAN population.
5. To determine whether selected biomarker levels measured at baseline and serially during follow-up are influenced by the use of immunosuppression.
6. To develop an RRS for Indian patients with IgAN.

## Sample size

Patients will be classified as being at low risk of progressive renal disease if their ARR score is <23 points or high risk if their ARR score of ≥23 points at baseline ^31^. Based on previous studies in India, we have assumed that the frequency of rapid progressors (>5 ml/min/1.73 m ^2^/year fall in eGFR) in the low-risk group will be 5%, and in the high-risk group will be 20% ^17, 23^. So, 176 patients give a power of 80% with an alpha error of 5% for detecting significant difference between proportions. Considering a 15% dropout rate (non-compliance to follow-up visits, unplanned pregnancies), 200 patients were recruited prospectively over 2.5 years (from March 2015 until the last quarter of 2017). We will look for significant differences among the exploratory biomarkers between the two risk groups and as independent variables to predict progressive decline in renal function and a composite end-point of at least 50% decline in eGFR and ESKD (eGFR <15 ml/min/1.73 m ^2^), renal replacement therapy (RRT) or death. We would like to find the best cut-off of the ARR score using received operating characteristic (ROC) curve methods. The best threshold will be obtained using likelihood ratio statistics, that is, by aiming to have high specificity with a high positive predictive value. However, the utility of the ROC will be decided based on the AUC. As part of the study apart from evaluating whether a different ARR threshold improves precision, we will also study whether addition of new biomarkers to an optimised Indian RRS improves the predictive value in an Indian population—this will include the C score of the new MEST-C score and exploratory biomarkers measured at baseline. At the same time, we will evaluate the accuracy in the Caucasian cohort and determine the additive value of additional measures to help predict Caucasian and Indian risk separately. In order to validate the usefulness of the threshold, another prospective validation cohort with a known threshold will be set up and followed to study the prognosis. However, in order to establish reliable CIs for the validity statistics bootstrap method (with 10,000 resampling) will be done in this cohort. We will include an equivalent number of healthy and disease controls to match the sample size of IgA patients. They will not be followed up longitudinally and will only be included in cross-sectional analyses at baseline.

## Study procedures and timeline

**Figure 2.**
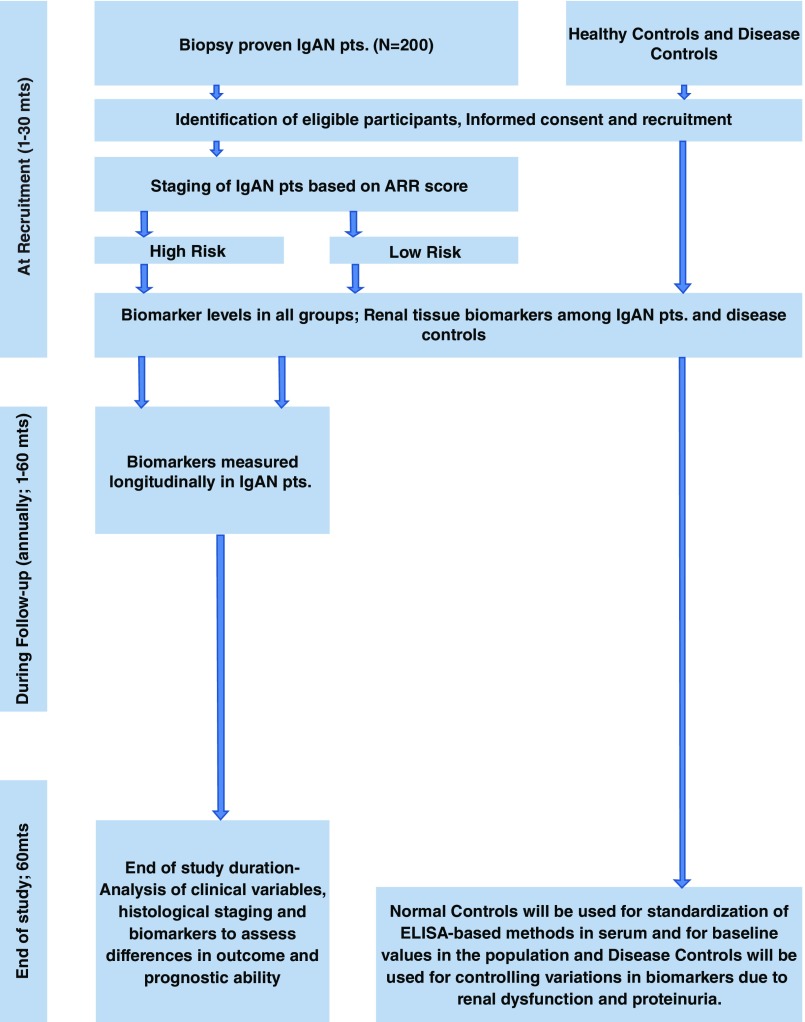
Flow diagram for recruitment and follow-up visits.

This is a prospective longitudinal cohort study with consecutive recruitment of incident IgAN patients. The baseline assessments will include the collection of demographic details, a physical examination, standard clinical laboratory tests and collection of biological samples (DNA, serum, plasma, renal tissue, urine and faeces) for future biomarker and microbiomic analysis ^46^. Patients will be followed at least annually (usually 2–3 visits per year) and at each visit standard laboratory testing, alongside the collection of biological samples for exploratory analysis, will be undertaken. The study co-ordinators will also contact patients telephonically or through postal/email correspondence to assess their adherence to medications, optimise the doses of RAS blockers according to the self-reported blood pressure and renal function tests. There will be a pre-specified interim analysis of the cohort once all patients have completed 2 years of follow-up. All patients will be followed-up for 5 years. Timelines for recruitment and follow-up visits are summarized in Figure 2.

## Statistical analysis

Demographic data on patients will be gathered and suitable comparisons will be made to ensure that the groups are well-matched. All outcome measures will undergo appropriate omnibus tests to investigate statistical differences between groups and within groups. Risk factors for progressive disease in an Indian population will be evaluated using a Cox proportional hazard model with a stepwise backward elimination method and then compared with those comprising the ARR score derived from a Japanese cohort. To create a renal prediction risk score specifically for Indian IgAN (either a modified ARR or completely new score), incorporating measures of exploratory biomarkers, the score for each variable will be weighted by the regression coefficients calculated using the relevant Cox model. In addition to this, we will look at associations between outcome measures and investigate potential interactions between variables to generate hypotheses for future studies. All statistical analyses will be done using SPSS version 21 and GraphPad (Prism) version 7.

## Patient and public involvement

This observational study has been designed based on the retrospective data from our institute ^22, 23^ and the felt need of all stakeholders for urgent research in this area. The patients and the public were not involved in the design or conduct of this study, but they are the intended benefactors of this research. The results will be disseminated electronically to all participants in the study.

## Strengths and limitations

Strengths of this protocol include the fact that this is a prospective longitudinal design with consecutive recruitment, reducing recruitment bias and enabling longitudinal biomarker evaluations. To our knowledge, this is also the first South Asian IgAN cohort to be established. In addition, bio-banking of clinical samples to a standardised protocol for future research will enable us to test emerging hypotheses on a well-established cohort.

Limitations of this protocol include the fact that pediatric (those aged <18 years) cases are excluded and limits the results interpretation to only adult patients. Additionally, participants with advanced kidney disease (eGFR <10 ml/min/1.73 m ^2^) are excluded so study conclusions may not reflect this subset of individuals.

## Ethics and dissemination

The full protocol was presented to the Institutional Review Board (Silver, Research and Ethics Committee) of the Christian Medical College, Vellore, India. Approval was provided vis. Ref. No. IRB Min. No. 8962 [Other] dated 23.07.2014 and Ref. No. IRB Min. No. 9481 [Other] dated 24.06.2015.

The study was submitted for external peer review as part of an application to the Wellcome Trust/DBT India Alliance and the lead author was awarded the Wellcome Trust/DBT India Alliance Early Career Fellowship [grant number IA/CPHE/14/1/501501].

Written informed consent has been obtained from participants in line with recommendations set out in E6(R1) of the International Council for Harmonisation of Technical Requirements for Pharmaceuticals for Human Use (ICH) Good Clinical Practice guidelines. Original consent forms will be stored, and records and data will be managed to ensure confidentiality per the recommendations of the Indian Council of Medical Research (ICMR) Ethical Guidelines for Biomedical Research on Human Subjects. Material transfer agreement to the collaborators’ laboratory has been approved by the ICMR.

## Dissemination plan

We plan to present all data at national and international conferences. Furthermore, we will also publish the findings in international journals. We expect to publish our initial findings during late 2018. Following the publication of our results, anonymised participant level data will be available for collaborations, on request. The GRACE-IgANI study is registered with the ISRCTN ( ISRCTN36834159) and the registration status is retrospective as patient recruitment started before registration.

## Data availability

All data underlying the results are available as part of the article and no additional source data are required.

## Supplementary material

**Supplementary File 1. Completed STROBE checklist for this study.**
