# Supplementary material for: Protocol and rationale for the first South Asian 5-year prospective longitudinal observational cohort study and biomarker evaluation investigating the clinical course and risk profile of IgA nephropathy: GRACE IgANI cohort
Source: Wellcome Open Res. 2018 Jul 26;3:91. [Version 1] doi: 10.12688/wellcomeopenres.14644.1 (PMC6148466; doi:10.12688/wellcomeopenres.14644.1)
Supplement: Peer review history

# Peer review: Referee response for version 1

**DOI:** 10.21956/wellcomeopenres.15946.r33587

**Published:** 2018-09-19

**Authors:** Colin C. Geddes

**Affiliations:** 1 The Glasgow Renal and Transplant Unit, Queen Elizabeth University Hospital, Glasgow, UK; 2 University of Glasgow, Glasgow, UK

The authors plan a longitudinal observational study of Indian patients presenting with IgA nephropathy (IgAN) correlating a range of established clinical and laboratory markers with renal function outcome to determine if the same prognostic factors apply to an Indian cohort as a caucasian cohort. This is novel and relevant because there are known epidemiological ethnic differences in IgAN as described in the proposal. 

 In addition they plan to correlate outcome with novel biomarkers that are already being studied in Caucasian cohort and this has the potential to improve understanding of the pathogenesis of IgAN and generate targets for therapy in high risk subjects.

 This adds to the work already being done by members of the group who have international expertise in studies of the pathogenesis and epidemiology of IgAN. 

 The background and methods are clearly stated.  I answered 'partly' to the question about the methods being presented sufficiently to be replicated simply because not all of the biomarkers to be assessed have been identified yet.

 There are many strengths to the proposed study. In particular the fact that one of the cohorts for study has already been recruited.  I was slightly confused about whether the subjects for the Indian cohort had already been recruited; the 'Patient recruitment' section implied they have not and the 'Sample Size' section implied they have. 

 One potential limitation is the effect that immunosuppression might have on the longitudinal outcomes both in terms of renal function and biomarkers. The authors highlight that use of immunosuppression for IgAN is common in India and more common than will be the case in the caucasian cohort. They will recruit immunosuppression naïve subjects at baseline but it is not clear how they will handle the potential impact of subsequent immunosuppression. 

 Overall I think this study has the potential to add greatly to current knowledge and to the work already being done by this research collaboration.

I have read this submission. I believe that I have an appropriate level of expertise to confirm that it is of an acceptable scientific standard.

# Peer review: Referee response for version 1

**DOI:** 10.21956/wellcomeopenres.15946.r33586

**Published:** 2018-08-08

**Authors:** Evangeline Pillebout

**Affiliations:** 1 INSERM 1149, Center of Research on Inflammation, Paris, France; 2 Nephrology unit, St Louis Hospital, Paris, France

The protocol is well written and I cannot wait to read the results of this study which will provide essential information in the field of IgA nephropathy.

 Nevertheless, the paper could be improved by detailing some points:

 If I understand correctly, 4 groups of patients will be compared: Indian patients with NIgA, Caucasian and East Asian patients with NIgA, Indian patients with primary nephropathy other than NIgA and healthy patients (kidney donors). As the first group of patients is well described, the other 3 lack details and in particular we should modify Figure 2 by distinguishing the 4 groups and their respective numbers.

 In addition, some patients will be treated, which ones? With which treatment? Again, there is a lack of detail since we imagine that it is mainly the high-risk patients who will be treated with a certain risk of bias and if many groups of treatments are applied, the risk of loss of power in the analysis will be important.

 The primary endpoint is unclear: what rate of decline in GFR? Define patients with good or bad evolution...

 Finally, a table summarizing the prognostic factors (biomarkers) which will be evaluated could be added.

I have read this submission. I believe that I have an appropriate level of expertise to confirm that it is of an acceptable scientific standard.
